# A multi-institutional phase I study of acetazolamide with temozolomide in adults with newly diagnosed *MGMT*-methylated malignant glioma

**DOI:** 10.1093/noajnl/vdae014

**Published:** 2024-02-01

**Authors:** Riley K Driscoll, Sean B Lyne, David J Voce, Stefania Maraka, Vinai Gondi, Steven J Chmura, Karan S Dixit, Priya U Kumthekar, Theodore G Karrison, Peter Pytel, John M Collins, Roger Stupp, Ryan T Merrell, Rimas V Lukas, Bakhtiar Yamini

**Affiliations:** Department of Neurological Surgery, University of Chicago Medicine, Chicago, Illinois, USA; Department of Neurological Surgery, University of Chicago Medicine, Chicago, Illinois, USA; Department of Neurological Surgery, Vanderbilt University Medical Center, Nashville, Tennessee, USA; Department of Neurology and Rehabilitation, University of Illinois at Chicago, Chicago, Illinois, USA; Proton Therapy Center and Northwestern Medicine Cancer Center, Warrensville, Illinois, USA; Department of Radiation and Cellular Oncology, University of Chicago Medicine, Chicago, Illinois, USA; Department of Neurology, Feinberg School of Medicine, Northwestern University, Chicago, Illinois, USA; Lou & Jean Malnati Brain Tumor Institute, Robert H. Lurie Comprehensive Cancer Center, Northwestern University, Chicago, Illinois, USA; Department of Neurology, Feinberg School of Medicine, Northwestern University, Chicago, Illinois, USA; Lou & Jean Malnati Brain Tumor Institute, Robert H. Lurie Comprehensive Cancer Center, Northwestern University, Chicago, Illinois, USA; Department of Public Health Sciences, University of Chicago, Chicago, Illinois, USA; Department of Pathology, University of Chicago Medicine, Chicago, Illinois, USA; Department of Radiology, University of Chicago Medicine, Chicago, Illinois, USA; Department of Neurology, Feinberg School of Medicine, Northwestern University, Chicago, Illinois, USA; Lou & Jean Malnati Brain Tumor Institute, Robert H. Lurie Comprehensive Cancer Center, Northwestern University, Chicago, Illinois, USA; Division of Hematology/Oncology, Department of Medicine, Feinberg School of Medicine, Northwestern University, Chicago, Illinois, USA; Department of Neurological Surgery, Feinberg School of Medicine, Northwestern University, Chicago, Illinois, USA; NorthShore University Health System, Evanston, Illinois, USA; Department of Neurology, Feinberg School of Medicine, Northwestern University, Chicago, Illinois, USA; Lou & Jean Malnati Brain Tumor Institute, Robert H. Lurie Comprehensive Cancer Center, Northwestern University, Chicago, Illinois, USA; Department of Neurological Surgery, University of Chicago Medicine, Chicago, Illinois, USA

**Keywords:** acetazolamide, clinical trial, drug repurposing, glioblastoma, temozolomide

## Abstract

**Background:**

A significant unmet need exists for the treatment of glioblastoma, *IDH-*wildtype (GBM). Preclinical work shows that acetazolamide sensitizes GBM to temozolomide (TMZ) by overcoming TMZ resistance due to BCL-3-dependent upregulation of carbonic anhydrase. Acetazolamide is Food and Drug Administration-approved for the treatment of altitude sickness. Drug repurposing enables the application of drugs to diseases beyond initial indications. This multi-institutional, open-label, phase I trial examined a combination of acetazolamide and TMZ in patients with *MGMT* promoter-methylated high-grade glioma.

**Methods:**

A total of 24 patients (GBM, *IDH*-wildtype = 22; Grade 4 astrocytoma, *IDH*-mutant = 1; Grade 3 astrocytoma, *IDH*-mutant = 1) were accrued over 17 months. All patients received oral acetazolamide (250 mg BID for 7 days increased to 500 mg BID for Days 8–21 of each 28-day cycle) during the adjuvant phase of TMZ for up to 6 cycles.

**Results:**

No patient had a dose-limiting toxicity. Adverse events were consistent with known sequelae of acetazolamide and TMZ. In the 23 WHO Grade 4 patients, the median overall survival (OS) was 30.1 months and the median progression-free survival was 16.0 months. The 2-year OS was 60.9%. In total 37% of the study population had high BCL-3 staining and trended toward shorter OS (17.2 months vs N.R., *P* = .06).

**Conclusions:**

The addition of acetazolamide is safe and tolerable in GBM patients receiving standard TMZ. Survival results compare favorably to historical data from randomized trials in patients with *MGMT* promoter*-*methylated GBM and support examination of acetazolamide in a randomized trial. BCL-3 expression is a potential biomarker for prognosis in GBM or for patients more likely to benefit from TMZ.

**Key Points**

- A phase I trial of acetazolamide and temozolomide in *MGMT*-methylated GBM.
- The primary endpoint of safety was achieved with encouraging survival data.
- Findings support randomized investigation of acetazolamide in *MGMT*-methylated GBM.

**Importance of the Study**

Preclinical work identified the upregulation of carbonic anhydrase as a mechanism for resistance to temozolomide chemotherapy driven by the proto-oncogene BCL-3, a transcriptional co-activator in conjunction with NF-κB. Following prior success using the carbonic anhydrase inhibitor acetazolamide to sensitize GBM to temozolomide in murine models, the current multi-institutional, phase 1 trial was performed to examine the safety of combination acetazolamide and adjuvant temozolomide in *MGMT* promoter-methylated GBM. Twenty-four patients were enrolled. Median OS for GBM, *IDH-*wildtype tumors was 29.3 months and 2-year OS was 59%. Repurposing acetazolamide for a subset of GBM is an approach that may improve the standard of care treatment without altering the adverse event profile. Also, the tolerability of acetazolamide suggests that it can be safely included in other therapeutic strategies that include temozolomide as the backbone agent.

Glioblastoma, *IDH-*wildtype (GBM) is the most common primary glial neoplasm and one of the most aggressive cancers in humans. Standard management for GBM involves maximal safe tumor resection followed by radiation therapy (RT) in association with concomitant and adjuvant temozolomide (TMZ) chemotherapy.^1^ For over 2 decades, attempts to improve patient survival with novel chemotherapeutic or biologic agents have failed to demonstrate success against GBM in randomized clinical trials.^2,3^ The only therapy shown to enhance the survival of patients treated with a combination of RT and TMZ has been a device exposing the brain continuously to alternating electrical fields (tumor-treating fields [TTFields]).^4^ These findings illustrate the challenge inherent in translating promising experimental results into successful clinical strategies. Nevertheless, the unique success seen with TMZ suggests that enhancing the effectiveness of this agent may be a fruitful approach to improving the management of this disease.

GBM is a heterogeneous malignancy comprised of molecular subtypes that not only have relevance to prognosis but also to their response to therapy.^5^ Methylation of the promoter of the O-6-methylguanine-DNA methyltransferase (*MGMT*) gene, a direct DNA repair enzyme, is the strongest predictor of response to TMZ in GBM.^6,7^ Although the median overall survival (OS) of GBM patients treated with combination RT/TMZ is approximately 15 months, in patients with a methylated *MGMT* promoter, OS is significantly higher.^8^

In addition to MGMT, other molecular biomarkers have been shown to separate GBM into prognostic groups.^9^ In this regard, expression of the proto-oncogene BCL-3, a nuclear factor-κB (NF-κB) co-regulator, was found to not only identify prognostic groups of GBM but also to potentially act as a predictor of response to alkylators like TMZ.^10^ This latter observation remains to be validated in a prospective study. Elevated BCL-3 was shown to promote resistance to TMZ by up-regulating carbonic anhydrase II (CAII). Accordingly, the addition of the CA inhibitor acetazolamide (ACZ) significantly increased the survival of mice bearing intracranial GBM xenografts with low MGMT protein abundance.^10^

Acetazolamide (ACZ) has been used clinically for over 50 years for numerous medical conditions including glaucoma, epilepsy, altitude sickness, and idiopathic intracranial hypertension. A clear oral dosing regimen has been established for ACZ to treat these conditions. Although ACZ inhibits multiple CA isoforms, it has high potency against CAII.^11^ The exact mechanism by which ACZ mediates its clinical effects is unclear. In general, by inhibiting CA, ACZ modulates the ratio of CO_2_, H_2_O, and bicarbonate ions. Continued use of ACZ is associated with tolerance requiring an increase in dose.^12^ Intermittent dosing, with dose escalation built into the schedule, represents a means of attempting to overcome drug tolerance and is an important novel aspect of the current study regimen. The potential of using ACZ as an anti-tumor agent has been hypothesized for many years with some success in peripheral tumors in mice.^13^ In addition, further support for targeting CA for the treatment of GBM is seen in the preclinical success of inhibitors of other CA isoforms.^14^

This phase I study was designed following xenograft studies that revealed a chemosensitizing effect of ACZ in experimental GBM that have a methylated *MGMT* promoter.^10^ Here, an initial cohort of 24 patients was enrolled with the primary outcome being safety and tolerability in patients with newly diagnosed, *MGMT* promoter-methylated GBM. The study analysis was undertaken after all patients had achieved a minimum of 24 months follow-up after trial enrollment.

## Materials and Methods

### Study Design

An open-label, single-arm, multi-institutional phase I study was performed to examine the safety and tolerability of adding ACZ to adjuvant TMZ in adults with newly diagnosed, *MGMT* promoter-methylated GBM or grade III astrocytoma (according to the WHO 2016 criteria).^15^ Participants were screened and enrolled at any point after diagnosis and prior to the initiation of adjuvant TMZ. ACZ was administered during the adjuvant TMZ phase for up to 6 cycles. The primary endpoint was the safety of a combination of ACZ and adjuvant TMZ in newly diagnosed, high-grade glioma patients. Secondary endpoints included OS, progression-free survival (PFS), and correlations between BCL-3 staining and survival. Accrual began on August 13, 2018, and the last patient was enrolled on January 28, 2020. Adverse events (AEs) were determined based on the National Cancer Institute Common Terminology Criteria for Adverse Events (CTCAE) v 4.03. The protocol was reviewed and approved by the Institutional Review Board of each participating center. All patients gave written informed consent for trial participation.

### Eligibility and Patient Characteristics

Eligible adult (>18 years) patients had a newly diagnosed, histologically confirmed, supratentorial GBM or high-grade glioma (WHO 2016 criteria)^15^ with a methylated *MGMT* promoter as assessed by the standard institutional technique. Subjects were deemed eligible if they had a Karnofsky performance status ≥60 and were to receive TMZ as part of the standard adjuvant treatment regimen following concomitant TMZ and RT. In addition, eligibility required the following pretreatment laboratory values: Absolute neutrophil count ≥1.5 × 10^9^/L; Platelets ≥ 100 × 10^9^/L; Hemoglobin ≥ 8.0 g/dL; creatinine level within normal institutional limit or creatinine clearance ≥ 60 mL/min/1.73 m^2^; Aspartate transaminase and alanine transaminase <2.5× institutional upper limit of normal (ULN); Total bilirubin ≤ 1.5 × ULN, INR within 1.5 × ULN (or if receiving anticoagulant therapy INR of ≤ 3.0 with concomitant increase in PT or an aPTT ≤ 2.5 × control) and negative pregnancy test within 30 days of registration if relevant. Participants were excluded from the study if they met any of the following criteria: prior invasive malignancy (except non-melanomatous skin cancer, carcinoma in situ of the cervix, or prior low-grade glioma) unless they had been disease-free and off therapy for that disease for a minimum of 3 years; active systemic infection requiring treatment including HIV infection or toxoplasmosis; other severe acute or chronic medical or psychiatric condition, or laboratory abnormality that may increase the risk associated with study participation or ACZ administration (e.g., acidosis, adrenocortical insufficiency, and cirrhosis); systemic corticosteroid therapy >8 mg of dexamethasone daily (or equivalent) at study enrollment; hypersensitivity to ACZ or sulfonamides.

### Treatment Agent and Dosing

**Figure 1.**
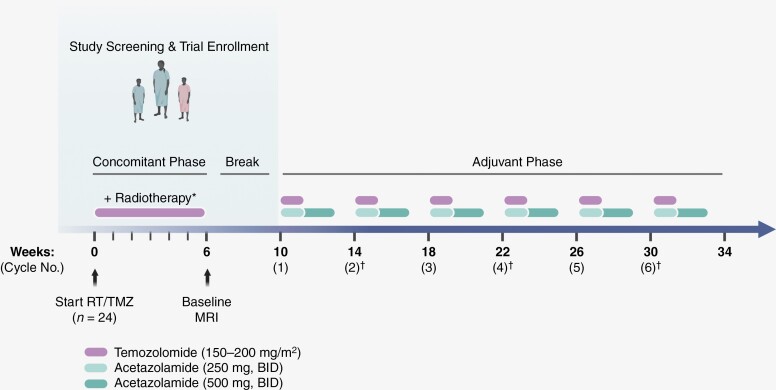
Schematic description of the ACZ-TMZ Trial. *Elderly patients (>65 y) were permitted to undergo an alternative 3-wk radiotherapy course during concomitant phase on an individual/elective basis. †Approximate serial 2-month MRI schedule.

Acetazolamide (ACZ) is a Food and Drug Administration-approved agent and was used in this trial with a Department of Health and Human Services Investigational New Drug exemption. ACZ was prescribed by individual study investigators and thus participants may have received either the original Diamox formulation or one of several generic ACZ variants. The most commonly noted side effects due to ACZ include dysesthesias and dysgeusia (metallic taste), especially on consumption of carbonated drinks. ACZ was started on Day 1 together with Day 1 of the first adjuvant TMZ cycle and given for 21 consecutive days beginning at a dose of 250 mg twice a day for the first 7 days, then escalated to 500 mg twice a day for days 8–21. ACZ administration was repeated in the same manner, including intra-cycle dose escalation, every 28 days synchronous with the start of the next adjuvant TMZ cycle. Adjuvant TMZ was prescribed per local standard practice and in accordance with its label. In Cycle 1, TMZ was to be dosed at 150 mg/m^2^ daily for 5 days. In the absence of myelotoxicity, the TMZ dose was to be escalated to 200 mg/m^2^ for 5 days for subsequent cycles (Figure 1). This dosing regimen was chosen to minimize the chance of tolerance to ACZ, a finding routinely appreciated clinically with continuous ACZ dosing at a single concentration.

### Primary and Secondary Endpoints

The primary endpoint was safety and tolerability as assessed by analysis of AEs graded per CTCAE v4.03 criteria. The study was designed to examine the rate of dose-limiting toxicity (DLT) following combination ACZ and TMZ. DLT was defined as any of the following treatment-related AEs that occurred within 28 days of treatment initiation including: Grade 4 non-hematological toxicity except alopecia, nausea, and vomiting, or lymphopenia; Grade 3 non-hematological toxicity that results in a delay of TMZ by >4 weeks; Grade 4 thrombocytopenia (<25 000/ mm^3^) that results in a delay of TMZ for >4 weeks and Grade 4 neutropenia (<500/ mm^3^) lasting >7 days, or Grade 3 febrile neutropenia (<1 000/ mm^3^).

Secondary endpoints of OS and PFS were calculated from the time of trial consent. OS was determined until the date of death and patients alive at the end of the study were censored on the date they were last known to be alive. Survival analyses were performed according to the Kaplan–Meier methods. Patients alive and free from progression at the time of analysis were censored at their last tumor assessment date. In addition, response to treatment was qualitatively assessed by an independent neuroradiologist based on the change in tumor size as determined by Response Assessment in Neuro-Oncology (RANO) criteria.^16^ Specifically, tumor dimensions on the 6-month MRI were compared to the baseline MRI.

BCL-3 protein abundance was determined by immunohistochemical analysis of formalin-fixed, paraffin-embedded (FFPE) surgical specimens. Specimens obtained at the time of initial surgery were embedded at the local institution and slides were shipped to the University of Chicago. Slides were incubated in antigen retrieval buffer (DAKO, S1699) and heated at >97 °C for 20 minutes. Anti-BCL-3 antibody (A02773-2, Boster, 1:200) was applied for 1 hour. Subsequently, sections were incubated with biotinylated anti-rabbit IgG (1:200, BA-1000, Vector Laboratories) for 30 minutes and the antigen-antibody binding detected by Elite kit (PK-6100, Vector Laboratories) and 3,3ʹ-diaminobenzidine (DAB) (DAKO, K3468) system. Scoring of BCL-3 staining was performed as previously described^10^ in a blinded fashion by 3 independent observers (R.K.D., D.J.V., and B.Y.) and a neuropathologist (P.P.). Only nuclear staining was considered positive and inter-observer differences were verified by re-examination of the individual specimen in a blinded manner. Scoring was performed in a semiquantitative fashion based on a 4-tier system: 0 (no staining), 1 (<25% positive cells), 2 (25%–75% positive), and 3 (>75% positive). This score was then converted into a binary grade in which a score of 0 or 1 was deemed low and a score of 2 or 3 was deemed high. BCL-3 staining grade was also correlated with OS.

### Sample Size Calculation and Statistical Analysis

A sample size of 24 patients was chosen based on a target DLT rate of <33% (chosen as a conservative estimate of toxicity associated with TMZ use in general clinical practice). An initial cohort of 12 patients was enrolled and if fewer than 4 patients (<33%) had a DLT, an additional 12 patients were recruited. The regimen was considered tolerable if less than 8 of 24 experienced DLT. Median OS and PFS were estimated using Kaplan–Meier Survival Analysis in IBM SPSS Statistics 29.

## Results

### Safety and Adverse Events

In this phase I trial, we examined the addition of ACZ to adjuvant TMZ in patients with newly diagnosed, high-grade glioma. Given the preclinical observation that ACZ is effective in tumors with a methylated *MGMT* promoter, enrollment was restricted to patients with *MGMT* promoter-methylated tumors regardless of *IDH* mutation status. Enrollment was completed in under 18 months. The 24 enrolled participants included 22 cases of GBM, *IDH-*wildtype (GBM), 1 WHO Grade 4 astrocytoma, *IDH*-mutant (*IDH-*mut), and 1 WHO Grade 3 astrocytoma, *IDH-*mut. Patient baseline characteristics are shown in Table 1. The median number of adjuvant cycles completed was 6 (range 0–6). Over 70% of patients completed at least 4 cycles of therapy. In 7 patients (29%) adjuvant TTFields were also delivered.

**Table 1.** Patient Demographics and Baseline Characteristics

Characteristics	Total (*n* = 24)
Sex, *n* (%)	
Male	14 (58.3)
Female	10 (41.7)
Age, median (range)	53 (31-85)
Race, *n* (%)	
White	19 (79.2)
Black	1 (4.2)
Declined/unknown	4 (16.7)
Ethnicity, *n* (%)	
Hispanic	2 (8.3)
Non-Hispanic	20 (83.3)
Declined/unknown	2 (8.3)
Initial KPS, *n* (%)	
90%–100%	18 (75)
70%–80%	5 (20.8)
60%	1 (4.2)
Median* (range)	95% (60–100)
Histopathological diagnosis^†^, *n* (%)	
Glioblastoma, *IDH*-wildtype	22 (91.7)
WHO Grade 4 astrocytoma, *IDH*-mutant	1 (4.2)
WHO Grade 3 astrocytoma, *IDH*-mutant	1 (4.2)
*IDH*-R132H mutational status, *n* (%)	
*IDH* wild-type	22 (91.7)
*IDH-*mutant^‡^	2 (8.3)
Extent of resection, *n* (%)	
Complete resection	8 (33)
Partial resection	15 (63)
Biopsy only	1 (4)
Enrollment phase, *n* (%)	
Prior to concomitant RT/TMZ	6 (25)
After RT/TMZ, prior to adjuvant TMZ	18 (75)
Protocol completion^§^, *n* (%)	
Completed protocol	14 (58.3)
Withdrew after **≥**5 cycles	2 (8.3)
Withdrew after 4 cycles	1 (4.2)
Withdrew after **≤**3 cycles	7 (29.2)
Reasons for premature withdrawal from study, *n* (%)	
Progressive disease	4 (16.7)
Adverse event^||^	3 (12.5)
Refused further treatment, unspecified	3 (12.5)
Tumor-treating fields (TTFields), *n* (%)	
−TTFields	17 (71)
+TTFields	7 (29)
Baseline corticosteroid use^¶^, *n* (%)	0 (0)

*Notes*: KPS = Karnofsky Performance Status.

*Median KPS among participants with WHO Grade 4 (*n* = 23) tumors is 90 (range = 60–100); median age and range are unchanged.

^†^Diagnosis made according to 2016 WHO classification guidelines for CNS tumors.

^‡^Among *IDH*-mutant tumors, 1 was a WHO Grade 3 astrocytoma and 1 was WHO Grade 4.

^§^Study protocol completion required 6 full TMZ-ACZ cycles.

^||^Adverse events leading to withdrawal included Stevens–Johnsons syndrome, confusion, and vague discomfort.

^¶^Considered >8 mg of daily corticosteroid use.

No patients experienced dose-limiting toxicity (primary endpoint and DLT). The observed treatment-emergent toxicities were attributable to either disease or known side effects of TMZ and ACZ. In a few instances, the dose of TMZ was held, but treatment was then resumed. Treatment-emergent AEs occurring in greater than 5% of patients are shown in Table 2.

**Table 2.** Adverse Events

Adverse events*, *n* (%)	Total (*n* = 24)	
	All Grades^†^	Grade 3 or 4
Any adverse events	23 (95.8)	6 (25)
General and constitutional events		
Nausea	12 (50)	—
Fatigue	10 (41.7)	—
Anorexia	9 (37.5)	—
Weight loss	5 (20.8)	—
Muscle weakness	4 (16.7)	—
Dehydration	3 (12.5)	—
Fall	3 (12.5)	—
Fever	2 (8.3)	—
Arthralgia	2 (8.3)	—
Allergic rhinitis	2 (8.3)	—
Hematologic events^‡^		
Thrombocytopenia	7 (29.2)	3 (12.5)
Hypokalemia	5 (20.8)	1 (4.2)
Neutropenia	4 (16.7)	4 (16.7)
Lymphocytopenia	3 (12.5)	2 (8.3)
Anemia	2 (8.3)	2 (8.3)
Low bicarbonate	2 (8.3)	—
Hyperchloremia	2 (8.3)	—
Gastrointestinal events		
Constipation	6 (25)	—
Diarrhea	5 (20.8)	—
Vomiting	5 (20.8)	—
Dysphagia	4 (16.7)	1 (4.2)
Dysgeusia	2 (8.3)	—
Xerostomia	2 (8.3)	—
Cardiovascular events		
Thromboembolic event	4 (16.7)	1 (4.2)
Hypertension	4 (16.7)	—
Extremity edema	2 (8.3)	—
Extremity pain	2 (8.3)	—
Genitourinary events		
Urinary frequency	5 (20.8)	—
Urinary tract infection	3 (12.5)	1 (4.2)
Dermatologic events		
Urticaria	2 (8.3)	—
Neurologic and psychiatric events		
Paresthesia	11 (45.8)	—
Confusion	7 (29.2)	2 (8.3)
Memory impairment	6 (25)	—
Seizure	5 (20.8)	1 (4.2)
Insomnia	5 (20.8)	—
Headache	4 (16.7)	—
Tremor	4 (16.7)	—
Agitation	3 (12.5)	—
Dysphasia	3 (12.5)	1 (4.2)
Dysesthesia	3 (12.5)	—
Gait disturbance	3 (12.5)	—
Aphasia	3 (12.5)	—
Tinnitus	2 (8.3)	—
Hallucinations	2 (8.3)	—
Cognitive disturbance	2 (8.3)	—

*Note*: *Data only include events occurring at >5% frequency for all adverse event grades.

^†^There were no recorded Grade 5 adverse events.

^‡^Among the aggregated cases of Grade 3 or 4 thrombocytopenia, 2 cases were Grade 3 and 1 was Grade 4. Among the aggregated cases of Grade 3 or 4 neutropenia, 2 cases were Grade 3 and 2 cases were Grade 4.

### Survival

**Figure 2.**
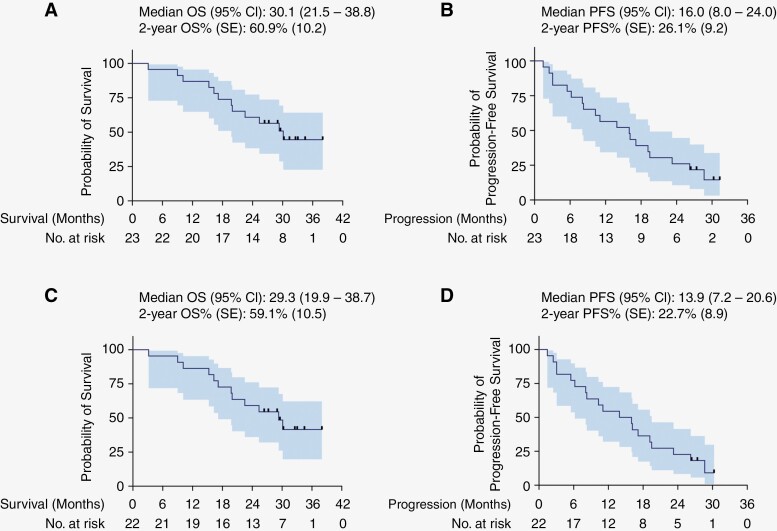
ACZ-TMZ and survival. Kaplan–Meier estimates of (A) overall and (B) progression-free survival among all WHO Grade 4 tumors. Kaplan–Meier estimates of (C) overall and (D) progression-free survival among GBM, *IDH*-wildtype tumors only. Tick marks correspond to censored observations. Shaded area corresponds to 95% confidence interval (CI). SE = standard error.

Survival was documented from the time of trial enrollment, which occurred anywhere from prior to concomitant TMZ/RT up to the point of adjuvant TMZ initiation (Figure 1). The median time between diagnosis and consent was 3.0 months. At the time of this analysis, 13 of 23 patients had succumbed to their disease and 4 patients remained progression-free. For patients with WHO Grade 4 tumors, median OS was 30.1 months (95% CI: 21.5–38.8; Figure 2A) with a 2-year survival rate of 60.9% (95% CI: 41.0–80.8). Median PFS was 16.0 months (95% CI: 8.0–24.0; Figure 2B). When restricting the analysis to GBM, *IDH-*wildtype tumors, median OS was 29.3 months (95% CI: 19.9–38.7; Figure 2C) with a 2-year survival rate of 59.1% (95% CI: 38.5–79.6). Among this same group, median PFS was 13.9 months (95% CI: 7.2–20.6; Figure 2D). Patients also receiving treatment with TTFields had a non-significant prolongation of PFS by 2.2 months and no difference in OS was demonstrated. (Supplementary Figure S1).

**Figure 3.**
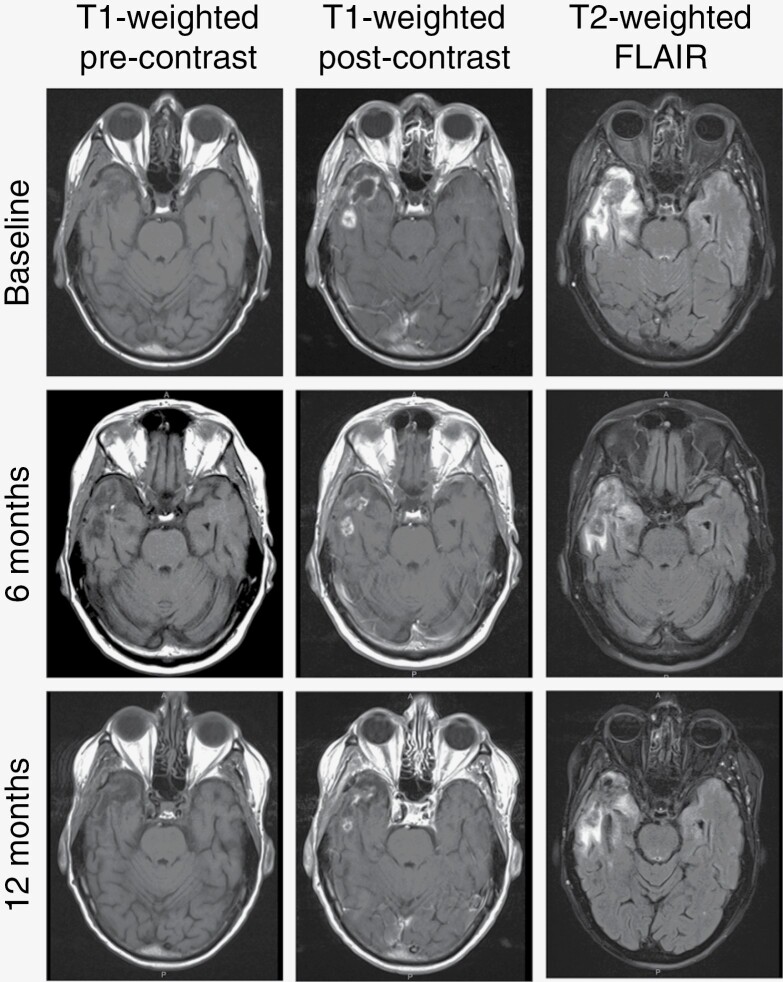
Radiographic demonstration of treatment response. Representative axial MRI sequences demonstrating partial treatment response at 6 and 12 months in a 68-year-old patient with a right temporal GBM, *IDH*-wildtype tumor. Baseline scan obtained approximately 4 months following diagnosis and after concomitant TMZ/RT.

To further evaluate the response to treatment, we examined MRI scans 6 months following initiation of ACZ. Tumor size on T1 contrast-enhanced scans was compared to that on studies obtained after concomitant TMZ/RT and prior to initiation ACZ/TMZ (Figure 1). Images were analyzed in a blinded manner by a neuroradiologist, and results were reported based on RANO criteria. Just over 60% of patients had stable disease or better with nearly 35% showing partial or complete response (Figure 3 and Supplementary Table S1).

### BCL-3

**Figure 4.**
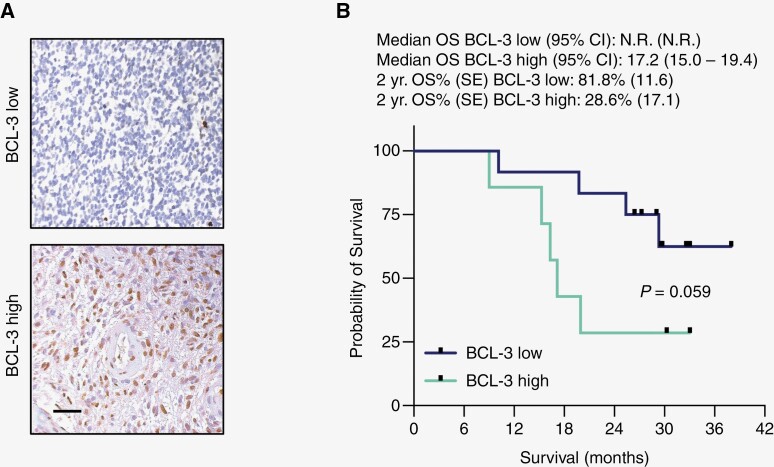
BCL-3-stratified survival. (A) Representative IHC staining of tumor specimens with anti-BCL-3 showing low- and high-grade staining as indicated (40× magnification, scale bar = 5 μm). (B) Kaplan–Meier estimates with log-rank comparison of overall survival in patients with WHO Grade 4 tumors separated by BCL-3 staining grade (high or low). Tick marks correspond to censored observations. *P* = .059. SE = standard error; N.R. = value not reported as more than half of the cohort failed to achieve an event.

Given the preclinical association between BCL-3 and response to TMZ, we examined the abundance of BCL-3 in FFPE slides obtained at the time of initial surgery. IHC was used to examine BCL-3 abundance in the nuclei of tumor cells and staining scored on a 4-point scale that was converted to a binary grade (high or low, Figure 4A). Nineteen patients (79.1%) had tissue available for IHC analysis. Of these, 7 tumors (37%) were found to have high nuclear BCL-3 staining. In a univariate analysis, patients with high BCL-3 trended toward shorter median OS than those with low BCL-3 staining (17.2 months vs N.R., respectively; Figure 4B, *P* = .06). Also, while 2-year OS was 82% in those with low BCL-3 abundance, in patients with high BCL-3, 2-year OS was only 29%.

## Discussion

In this phase I clinical trial, we examined the safety and tolerability of adding the oral CA inhibitor ACZ to standard adjuvant TMZ in patients with *MGMT* promoter-methylated GBM and high-grade glioma. Analysis of the primary endpoint revealed that the combination of ACZ and TMZ was well tolerated with no dose-limiting toxicity. Mild and anticipated side effects previously described with ACZ were observed including dizziness, anorexia, tingling sensations, and abnormal taste. TMZ-associated side effects were also observed at the expected rate.

Analysis of secondary outcome endpoints was restricted to either GBM *IDH-*wildtype only or to patients with WHO Grade 4 tumors (*IDH-*wildtype or *IDH-*mutant, according to the 2021 WHO classification). Survival was determined from the consent date. Although the majority of patients consented after RT, 25% of the cohort was enrolled prior to RT (Table 1). This wide enrollment window introduces some artificial variability into survival due to trial design. Among GBM *IDH-*wildtype participants, median OS was 29.3 months, while PFS was 13.9 months, and survival at 2 years was over 59%. These data compare favorably to findings from contemporary randomized clinical trials performed in patients with *MGMT* promoter-methylated GBM. For example, in the initial landmark study examining TMZ in *MGMT*-methylated GBM, OS and PFS were 21.7 and 10.3 months, respectively, as measured from the time of randomization.^6,7^ In the *CENTRIC* trial, which calculated survival prior to initiation of RT, among *MGMT* promoter-methylated GBM patients median OS was 26.3 months while 2-year OS was 56%.^2,3^ In the recent CeTeG/NOA-09 study that examined the addition of CCNU to TMZ, in patients with *MGMT* promoter-methylated GBM, median OS calculated from before RT was 30.4 months in the TMZ arm.^17^ Importantly, these comparisons must be considered in the context of the enrollment strategies used in each study. In the aforementioned studies, randomization occurred prior to RT, whereas studies such as the present trial, which enrolled patients predominantly after RT, potentially exclude patients with more aggressive tumors who might have succumbed to their disease early. Despite these reservations, the current data remain favorable in comparison to trials that enrolled patients after completion of RT. For example, in the EF-14 trial that identified TTFields as a potential standard therapy for GBM, OS was 21.2 months in *MGMT* promoter-methylated patients who received TMZ and RT alone.^4^ Moreover, in the international study investigating dose-dense TMZ, among *MGMT* promoter-methylated patients receiving standard dose TMZ, OS and PFS were 21.4 and 6.5 months, respectively.^18^

In addition to survival, secondary endpoints also included a 6-month radiographic response assessment and examination of BCL-3 protein in biopsied specimens. In regard to the former, 35% of GBM patients demonstrated a response at 6 months (Supplementary Table S1). Although this finding is encouraging as TMZ itself does not routinely lead to objective imaging response, the data may overestimate the response due to the presence of tumor pseudoprogression.^19^ Given that baseline MRIs in this trial were performed soon after concomitant TMZ/RT, signal reduction on ensuing scans may represent the resolution of transient enhancement changes as opposed to genuine treatment response, a finding bolstered by the observation that pseudoprogression is higher in *MGMT* promoter-methylated tumors.^20,21^ On the other hand, methylated tumors are also more likely to demonstrate stability or response on initial post-RT imaging.^22^ In sum, these findings support further controlled analysis of imaging outcomes in future studies. With respect to BCL-3 staining, approximately one-third of patients were found to have high nuclear BCL-3 in tumor biopsies as assessed by immunostaining. This value was consistent with prior work in archived GBM samples.^10^ A trend toward greater survival was seen in the patients with low BCL-3 protein staining, a finding also noted in the prior work. Moreover, the median OS in patients with high nuclear BCL-3 (17.2 months) is substantially lower than that of patients with *MGMT* promoter-methylated GBM from prior clinical trials. Although these data are interesting and support a potential role for BCL-3 as a biomarker in GBM, conclusions regarding the role of BCL-3 in outcome will require further work.

The current study was designed following the results of preclinical work in which TMZ-induced upregulation of CA in GBM with low MGMT protein, or a methylated *MGMT* promoter, via a mechanism involving BCL-3-mediated resistance to TMZ.^10^ Given that ACZ blocks CA activity, it was hypothesized that the addition of ACZ could sensitize tumor cells to TMZ-induced cell death. While such a chemosensitizing mechanism was the impetus for the trial, it is possible that ACZ also acts via other pathways to modify the response to TMZ and patient outcome. For example, ACZ may alter the local cerebral blood flow, thereby enabling increased TMZ delivery to the tumor environment.^23^

The use of ACZ for the treatment of GBM represents a potential drug repurposing strategy. Given the expense and time required to develop new agents for cancer therapy, drug repurposing is an approach that has significant advantages, including the ease and speed with which potential agents can be examined clinically.^24^ In the case of ACZ, its safety and tolerability were not unexpected given that this agent is broadly indicated for numerous medical conditions, including epilepsy and elevated intracranial pressure, sequelae routinely seen in patients with GBM. In addition to these advantages, repurposing a well-established drug for a new indication also has limitations. For example, repurposed compounds may lose their exclusivity, thus development for other indications may not receive the same level of industry investment as a novel, intellectual property-protected compound.

Taken together, the data from the current trial suggest that adding ACZ to TMZ is safe and does not add significant toxicity to standard adjuvant TMZ. Additionally, the survival data are comparable with the best historical data from patients with *MGMT* promoter-methylated GBM who were treated with standard therapy in prior randomized trials. This observation, together with the safety and ease of administration of ACZ supports a more definitive examination of this regimen in a randomized controlled manner. Notably, while ACZ was used only in the adjuvant phase of TMZ in the present trial, it is possible that earlier ACZ incorporation, during the concomitant TMZ phase, may have additional beneficial effects. In addition, the mechanism of action of ACZ suggests that its incorporation into more complex combination therapies that include TMZ, such as with TTF or immunotherapy, are strategies that also warrant study in the future.

## Supplementary Material

vdae014_suppl_Supplementary_Figures_S1_Tables_S1
