# Supplementary material for: A multi-institutional phase I study of acetazolamide with temozolomide in adults with newly diagnosed MGMT-methylated malignant glioma
Source: Neurooncol Adv. 2024 Feb 1;6(1):vdae014. doi: 10.1093/noajnl/vdae014 (PMC10901541; doi:10.1093/noajnl/vdae014)
Supplement: vdae014_suppl_Supplementary_Figures_S1_Tables_S1 [file vdae014_suppl_supplementary_figures_s1_tables_s1.docx]

**Supplementary Materials**

**A Multi-institutional Phase I Study of Acetazolamide with Temozolomide in Adults with Newly Diagnosed MGMT-methylated Malignant Glioma**

Riley K. Driscoll, Sean B. Lyne, David J. Voce, Stefania Maraka, Vinai Gondi, Steven J. Chmura, Sean Grimm, Karan S. Dixit, Priya U. Kumthekar, Theodore Karrison, Peter Pytel, John Collins, Roger Stupp, Ryan Merrell, Rimas V. Lukas, Bakhtiar Yamini*

*Corresponding author. Email: byamini@bsd.uchicago.edu

**Table of Contents: Supplementary Tables and Figures**

1. Figure S1. TTFields-stratified survival and progression.
2. Table S1. Six-month landmark treatment response.

**FIGURE S1.**

**Fig. S1. TTFields-stratified survival and progression**. Kaplan-Meier estimates with log-rank comparison of (A) overall and (B) progression-free survival among participants receiving TTFields compared to those who did not. Tick marks correspond to censored observations. Inset *P*-values represent output of log-rank analysis. Abbreviations: N.R. = value not reported. TTFields = tumor-treating fields.

**TABLE S1.**

**Table S1. Six-month landmark treatment response.**

| **Response** | **All patients**  **(*n* = 24)** | **All WHO Grade 4**  **(*n* = 23)** | **GBM, *IDH-*wildtype (*n* = 22)** |
| --- | --- | --- | --- |
| **Overall response^*^, *n* (%)** |  |  |  |
| Complete response | 1 (4.2) | 1 (4.3) | 1 (4.5) |
| Partial response | 7 (29.2) | 7 (30.4) | 7 (31.8) |
| Stable disease | 7 (29.2) | 6 (26.1) | 5 (22.7) |
| Progressive disease | 9 (37.5) | 9 (39.1) | 9 (40.9) |
|  |  |  |  |
|  |  |  |  |

**^*^Response assessed at 6 months after baseline, post-RT MRI per RANO criteria**
